# Characterization of the substrate binding site of an iron detoxifying membrane transporter from *Plasmodium falciparum*

**DOI:** 10.1186/s12936-021-03827-7

**Published:** 2021-06-30

**Authors:** Pragya Sharma, Veronika Tóth, Edel M. Hyland, Christopher J. Law

**Affiliations:** School of Biological Sciences, Queen’s University Belfast, 19 Chlorine Gardens, Belfast, BT9 5DL UK

**Keywords:** Iron homeostasis, Membrane transporter, Integral membrane protein, Vacuolar iron storage, Cytotoxicity, Transition metal cations, Comparative protein modelling, Substrate binding

## Abstract

**Background:**

*Plasmodium* species are entirely dependent upon their host as a source of essential iron. Although it is an indispensable micronutrient, oxidation of excess ferrous iron to the ferric state in the cell cytoplasm can produce reactive oxygen species that are cytotoxic. The malaria parasite must therefore carefully regulate the processes involved in iron acquisition and storage. A 273 amino acid membrane transporter that is a member of the vacuolar iron transporter (VIT) family and an orthologue of the yeast Ca^2+^-sensitive cross complementer (CCC1) protein plays a major role in cytosolic iron detoxification of *Plasmodium* species and functions in transport of ferrous iron ions into the endoplasmic reticulum for storage. While this transporter, termed PfVIT, is not critical for viability of the parasite evidence from studies of mice infected with VIT-deficient *Plasmodium* suggests it could still provide an efficient target for chemoprophylactic treatment of malaria. Individual amino acid residues that constitute the Fe^2+^ binding site of the protein were identified to better understand the structural basis of substrate recognition and binding by PfVIT.

**Methods:**

Using the crystal structure of a recently published plant VIT as a template, a high-quality homology model of PfVIT was constructed to identify the amino acid composition of the transporter’s substrate binding site and to act as a guide for subsequent mutagenesis studies. To test the effect of mutation of the substrate binding-site residues on PfVIT function a yeast complementation assay assessed the ability of overexpressed, recombinant wild type and mutant PfVIT to rescue an iron-sensitive deletion strain (*ccc1∆*) of *Saccharomyces cerevisiae* yeast from the toxic effects of a high concentration of extracellular iron.

**Results:**

The combined in silico and mutagenesis approach identified a methionine residue located within the cytoplasmic metal binding domain of the transporter as essential for PfVIT function and provided insight into the structural basis for the Fe^2+^-selectivity of the protein.

**Conclusion:**

The structural model of the metal binding site of PfVIT opens the door for rational design of therapeutics to interfere with iron homeostasis within the malaria parasite.

**Supplementary Information:**

The online version contains supplementary material available at 10.1186/s12936-021-03827-7.

## Background

Iron is an indispensable micronutrient for almost all species in the six kingdoms of life [1]. The unicellular protozoan parasites that cause malaria are no exception and are wholly dependent on their host as a source of the vital metal [2]. The redox properties of iron that enable it to readily cycle between the predominant ferric Fe^3+^ and ferrous Fe^2+^ oxidation states underpin biochemical reactions that function in a variety of cellular processes including those involved in energy production, respiration, and DNA replication [1, 3]. However, those same redox properties that have been exploited by organisms for beneficial purposes, and that make iron an essential constituent of many biological macromolecules, also render it potentially cytotoxic: oxidation of excess ferrous iron to the ferric state in the cell cytoplasm by Fenton/Haber–Weiss chemistry results in production of reactive oxygen species that are injurious to nucleic acids, lipids and proteins [4]. To prevent such cytotoxicity but at the same time ensure an adequate supply of essential iron, cells have evolved integrated mechanisms for maintenance of iron homeostasis through a carefully choreographed regulation of the systems that control iron acquisition and storage [1]. Cellular iron acquisition is commonly mediated by the activities of membrane transporters or receptor-mediated endocytosis [5–7]. Most bacteria, archaea, plant and animal cells accomplish intracellular iron storage by binding excess cytosolic Fe^2+^ to ferritin, a protein that oxidises the ferrous iron and stores it in an essentially unreactive state [1, 8]. However, some organisms do not express cytosolic ferritin and instead detoxify the cell cytoplasm by storing excess iron in vacuoles [1]. In yeast, for example, the movement of ferrous ions into the vacuole is facilitated by activity of the vacuolar membrane-bound Ca^2+^-sensitive cross complementer protein, CCC1 [9, 10]. Plants synthesize CCC1 homologues—integral membrane polypeptides of between 250 and 400 amino acids that belong to the vacuolar iron transporter (VIT) family of proteins—that localize Fe^2+^ to the vacuole [11–19]. Homologues of plant VITs have been identified in other eukaryotes (but, significantly, not in animals), bacteria and archaea [20]; and an orthologue of plant VIT is encoded by the genomes of several apicomplexan parasites, including those involved in pathogenesis of human disease states such as toxoplasmosis, cryptosporidiosis, and malaria [20, 21].

The malaria parasite has evolved a complex life cycle that constantly alternates between vertebrate hosts and anopheline mosquito vectors. Within human hosts, *Plasmodium* species are obligate intracellular parasites that transition through three different life cycle stages, including liver and erythrocytic stages during which the parasite must manage potentially toxic concentrations of iron [22]. The asexual intra-erythrocytic stage of the parasite life cycle offers a particular challenge to iron homeostatic processes due to release of labile Fe^2+^ into the cell cytosol by proteolytic digestion of host haemoglobin [23] and the cytosolic concentration of labile Fe^2+^ increases as the parasite matures from ring to schizont form [24]. A VIT orthologue plays a major role in cytosolic iron detoxification of *Plasmodium* species [21]. Although this transporter is not critical for viability of the parasite, and it is expressed during all life cycle stages, experiments on mice infected with VIT-deficient *Plasmodium* showed a significant reduction in parasite load in both liver and blood stages of infection compared to mice infected with wild type parasites [21, 25]. These data suggest PfVIT could provide an efficient target for chemoprophylactic treatment of malaria.

The most virulent malaria parasite of humans [26], *Plasmodium falciparum*, expresses a 273 amino acid, ~ 31 kDa plant VIT orthologue, named PfVIT, that functions in cytoplasmic iron detoxification by transport of ferrous ions, via Fe^2+^/H^+^ exchange, into the endoplasmic reticulum [21, 27]. In contrast to plant VITs and yeast CCC1, which have broader divalent metal cation specificity, PfVIT appears to function specifically as a Fe^2+^ transporter [21, 27]. To better understand the structural basis of substrate recognition and binding by PfVIT, the individual amino acid residues that constitute the Fe^2+^ binding site of the protein were identified. This was aided by a recently determined x-ray crystal structure of the vacuolar iron transporter *Eg*VIT1 from the plant *Eucalyptus grandis* with metal ion substrate bound [28]. Using the structure of *Eg*VIT1 as a template, a high-quality homology model of PfVIT was constructed to identify the amino acid composition of the transporter’s substrate binding site and to act as a guide for subsequent mutagenesis studies. To test the effect of mutation of the putative substrate binding-site residues on PfVIT function a yeast complementation assay was used to assess the ability of overexpressed, recombinant wild type and mutant PfVIT to rescue an iron-sensitive deletion strain (*ccc1∆*) of *Saccharomyces cerevisiae* from the toxic effects of high concentrations of Fe^2+^ [9]. This combined in silico and mutagenesis approach enabled identification of individual amino acid residues essential for coordination of Fe^2+^ to the PfVIT metal binding domain and speculation about the structural basis for the Fe^2+^-specificity of the transporter.

## Methods

All chemicals and reagents were Sigma-Aldrich brand purchased from Merck (UK) unless stated otherwise.

### Homology modelling

Initially, a number of homology models of PfVIT protein were built using the automated comparative modelling servers ModWeb [28], SWISS-MODEL [29] and I-TASSER [30]. The 273 amino acid primary sequence of PfVIT from the 3D7 isolate of *P. falciparum* (PlasmoDB ID: PF3D7_1223700) was used as the target input and three-dimensional crystal structures of *Eg*VIT1, a 234-residue protein from the plant *Eucalyptus grandis* (PDB IDs: 6IU3 and 6IU4) [31], were used as structural templates. The best-scoring models from each server were selected for further filtering and subjected to additional validation analyses using PROCHECK [32] and WHAT IF [33], and visualized by the PyMOL Molecular Graphics System, Version 1.8.4.0 (Schrödinger, LLC). On the basis of these quality tests, the best-performing model was judged as one generated by SWISS-MODEL that used the 3.5 Å crystal structure of *Eg*VIT1 (PDB code 6IU4) as the template.

A separate model of isolated cytoplasmic metal binding domain (MBD) of PfVIT, which consisted of 79 residues (Ala101-Leu179), was generated with MODELLER 9.24 [34] and the EasyModeller 4.0 GUI [35] using the 3.0 Å structure of the MBD fragment of *Eg*VIT1 bound to Fe^2+^ (PDB: 6IU9) as the template. The initial best model was further optimised, refined and energy minimised in MODELLER. Statistical quality assessment of the model was performed using PROCHECK [32]. The PfVIT MBD homology model was then superposed onto the isolated *Eg*VIT1 MBD crystal structure using the ‘align’ command of PyMOL. The atomic coordinates of the bound Fe^2+^ ion from the superposed *Eg*VIT1 MBD structure were subsequently extracted and transferred to the PfVIT MBD homology model to yield a final model of PfVIT cytoplasmic MBD bound to Fe^2+^ ion. Binding of Fe^2+^ to the final model was analysed using the CheckMyMetal (CMM): Metal Binding Site Validation Server [36] to identify individual amino acid residues that could potentially coordinate Fe^2+^ and form the binding site.

### Gene synthesis and site-directed mutagenesis

Previous work demonstrated that full-length *Plasmodium* VIT was expressed at low levels and was functionally impaired in a yeast heterologous expression system. In contrast, N-terminal truncation versions of the transporter demonstrated increased expression levels and activity and were easily detectable in vacuolar membrane fractions of the same system [21]. Therefore, an N-terminal truncation mutant of PfVIT, designated sPfVIT, was designed for this study. The 711 bp sequence encoding the 237 amino acid, D*2-36* N-terminal truncation mutant of the VIT from *P. falciparum* 3D7 (PlasmoDB gene ID: PF3D7_1223700) was codon-optimized for expression in *S. cerevisiae* and synthesized using a commercially available service (GenScript, USA). To facilitate detection of expressed protein and future protein purification, an in-frame thrombin cleavage site, *myc* epitope and His_10_ affinity tag were engineered into the C-terminal end of the protein to give a final construct of 822 bp (see Additional File 1: Figure S1). *BamH*1 and *Xho*1 restriction sites were introduced into the 5′ and 3′-ends of the synthetic DNA, respectively, and the codon optimized sequence was ligated into the multiple cloning site of pESC-Leu expression vector (Agilent, UK) that contained the *LEU2* selectable marker. This gave rise to pESC-Leu-sPfVIT that encoded a 273 amino acid construct of molecular mass 30.9 kDa, expression of which was placed under control of the *GAL1* promoter. Site- directed mutants of sPfVIT used in this study were engineered using a QuikChange II XL Site-Directed Mutagenesis Kit (Agilent, UK) according to the manufacturer’s protocol with the DNA primers listed in Additional File 2: Table S1, and with pESC-Leu-sPfVIT as template DNA. The fidelity of all constructs was verified by DNA sequence analysis (Macrogen Europe, Amsterdam). Plasmids were propagated in *Escherichia coli* XL10-Gold Ultracompetent cells (Agilent, UK) using carbenicillin for selection, then purified, quantitated and conserved at – 20 °C until required.

### Transformation of *Saccharomyces cerevisiae*

The budding yeast *S. cerevisiae* BY4741 derivative strain (*MAT*a *his3*Δ*1 leu2*Δ*0 met15*Δ*0 ura3*Δ*0*, *ccc1*Δ::*Kan*MX) that lacked CCC1 vacuolar iron transporter was used as the model organism for this study. The recipient yeast strain was transformed using a previously published method [37] except incubation temperatures were adjusted to 30 °C. Electrocompetent cells (45 µl aliquots) were transferred into pre-cooled sterile 1.5 ml Eppendorf tubes and 1–3 µg of DNA (either ‘empty’ pESC-Leu vector, wild type pESC-Leu-sPfVIT or mutant pESC-Leu-sPfVIT plasmid) added. The cells were then incubated on ice for 10 min prior to transfer to electroporation cuvettes. A single 1.5 kV, 200 Ω, 25 μF pulse was applied to the mixture using a MicroPulser Electroporator (Bio-Rad, UK) then 950 μl of YPD media (1% w/v Bacto™-yeast extract, 2% w/v Bacto™ -peptone, 2% w/v D-glucose) pre-warmed to 30 °C was added immediately into each cuvette. Cells were regenerated by incubation for 1 h at 30 °C then harvested by centrifugation (2200×*g*, 5 min). Harvested cells were resuspended in 100 μl of sterile water, plated on solid synthetic complete media lacking leucine (SC-Leu; 6.68 g/l yeast nitrogen base, 1.4 g/l yeast synthetic drop-out medium supplements without leucine, 20 g/l select agar, 2% w/v d-glucose) for selection and incubated for 2 days at 30 °C. Transformants were subsequently used for production of yeast cultures that overexpressed *P. falciparum* VIT.

### Overexpression of sPfVIT

Single colonies of each transformant were cultured in liquid SC-Leu medium containing 2% (w/v) D-glucose and incubated overnight at 30 °C with 180 rpm shaking. The overnight culture was diluted with SC-Leu to an OD_600_ of 0.2 in a sterile conical flask and further incubated until OD_600_ of 1.0. Protein expression was induced by addition of d-galactose to a final concentration of 3.5% (w/v) and the cultures grown for a further 20 h at 30 °C with shaking at 180 rpm. At the end of the induction period, samples of culture were taken for western blot analysis of protein expression levels and for use in qualitative and quantitative complementation assays designed to assess the ability of overexpressed protein to rescue the iron-sensitive *ccc1*∆ strain of *S. cerevisiae* from the cytotoxic effects of high concentrations of Fe^2+^.

### Isolation of vacuoles from *Saccharomyces cerevisiae*

Isolation of vacuoles from *ccc1Δ S. cerevisiae* cells that harboured ‘empty’ pESC-Leu vector (negative control cells) or that overexpressed wild type sPfVIT transporter from pESC-Leu-sPfVIT was performed based on a method described previously [38]. 500 ml of galactose-induced yeast culture was centrifuged at 3000×*g* for 5 min at room temperature. Harvested cells were resuspended in 50 ml of wash buffer (0.1 M Tris–HCl pH 9.4, 10 mM dithiothreitol) by gentle vortexing. The suspension was then incubated at 32 °C for 10 min then centrifugated at 2240×*g* for 6 min. The resulting pellet was resuspended in spheroplasting buffer (0.75 mM KH_2_PO_4_, 0.2% SC-Leu medium, 0.09 M sorbitol) containing 1 tablet of cOmplete™ EDTA-free Protease Inhibitor Cocktail (Roche) to a volume that contained 2 × 10^9^ cells/ml. Next, 50 U of Zymolyase 20T (MP Biomedicals, UK)/g wet weight of yeast cells was added and the mixture was incubated at 32 °C for 30 min with occasional swirling prior to centrifugation at 1000×*g* for 2 min at 4 °C. Spheroplasts were resuspended by gentle vortexing in 2.5 ml of 15% Ficoll solution (10 mM Pipes-KOH pH 6.8, 0.2 M sorbitol, 150 g/l Ficoll). An appropriate volume of Dextran solution (the µl volume of which was calculated by taking the final OD_600_ of a culture after 20 h of galactose induction × culture volume × 0.15) was added and the suspension held on ice for 2 min with occasional swirling. The suspension was then incubated at 32 °C for 3 min and placed back on ice. The spheroplasts were carefully layered onto a density step gradient consisting of 0%, 4%, 8% and 15% Ficoll solutions. The gradient was centrifuged at 175,000×*g* for 90 min at 4 °C and the vacuoles carefully harvested from the 0 to 4% Ficoll interface. The total protein content of the recovered vacuoles was quantified subsequent to analysis by western blot.

### Yeast complementation assays

Cultures from single colonies of the *ccc1*∆ strain of *S. cerevisiae* transformed with either ‘empty’ pESC-Leu vector, or pESC-Leu that encoded wild type or mutant sPfVIT, were grown and protein expression induced as described above. At the end of the induction period the cultures were diluted to OD_600_ of 0.2 with SC-Leu medium. The cultures were then twofold serially diluted and 10 µl of each spotted onto solid medium induction plates (SC-Leu agar containing 3.5% w/v galactose and 2 mM ascorbic acid) with/without 7.5 mM ammonium iron (II) sulphate. The ascorbic acid and ammonium iron (II) sulphate solutions were freshly prepared then argon flushed to minimize oxidation of the ferrous iron during handling. Plates were incubated at 30 °C for 3 days prior to imaging with an Azure Biosystems C200 gel documentation imager (Cambridge Bioscience, UK).

A quantitative analysis of the ability of wild type and mutant sPfVIT to rescue the *ccc1*∆ strain of *S. cerevisiae* was performed using a colony count assay. Cells were grown and protein expression induced as described above. At the end of the induction period samples of each culture were taken and diluted with SC-Leu medium to OD_600_ of 0.002. Aliquots (50 µl) of diluted cultures were spread onto Petri dishes that contained solid media (SC-Leu agar, 3.5% w/v galactose, 2 mM ascorbate) with/without 7.5 mM ammonium iron (II) sulphate. The plates were incubated at 30 °C for 3 days then individual colonies counted. All assays were performed in triplicate. The number of colonies formed per ml of OD_600_ 1.0 was calculated for each and the data analysed using one-way analysis of variance (ANOVA) and Dunnett’s multiple comparison tests.

### Protein quantitation and western blot analysis

Total protein in yeast whole cell samples and in vacuoles was determined by Pierce BCA Protein Assay Kit (ThermoFisher Scientific, UK) used according to the manufacturer’s instructions. Samples of yeast cell cultures that had sPfVIT expression induced for 20 h were prepared for SDS-PAGE by adjustment of OD_600_ to 1.0 in a volume of 1 ml with ultrapure H_2_O. Cells were pelleted by centrifugation, resuspended in 200 μl of 0.2 M NaOH and incubated at RT for 10 min prior to addition of 75 μl of 5× concentrated SDS-PAGE sample loading buffer (300 mM Tris–HCl pH 6.8, 50% v/v glycerol, 5% w/v SDS, 0.05% v/v bromophenol blue, 250 mM dithiothreitol). Samples containing 30 µg of total protein (for vacuole samples 7 µg of total protein was added) were electrophoresed on a 10-well Novex WedgeWell 4 to 20% Tris–Glycine gel (Invitrogen, UK) in Tris–Glycine SDS running buffer at constant 200 V for 45 min. Proteins were then transferred from the gel onto a 0.22 µm nitrocellulose membrane (MDI Membrane Technologies, USA) at constant 30 V for 16 h at 4 °C using a Mini Trans-Blot apparatus (Bio-Rad, UK). After transfer the membrane was blocked for 1 h at RT in TBS-T buffer (50 mM Tris pH 7.5, 0.01% v/v Tween 20, 150 mM NaCl) containing 1.0% w/v BSA then washed twice in TBS-T buffer. For detection of the His_10_ tag of the recombinant transporter the membrane was probed at RT for 1 h with HisProbe-HRP conjugate (ThermoFisher Scientific, UK) diluted 1:5000 in TBS-T buffer pre-treated with 0.1% w/v BSA. The membrane was then washed four times in TBS-T buffer prior to detection of the probe with SuperSignal West Pico PLUS Chemiluminescent Substrate (ThermoFisher Scientific, UK). The membrane was subsequently imaged with a G:BOX Chemi XRQ gel doc system (Syngene, USA).

## Results

### Three-dimensional structural model of *Plasmodium falciparum* VIT (PfVIT)

**Fig. 1:**
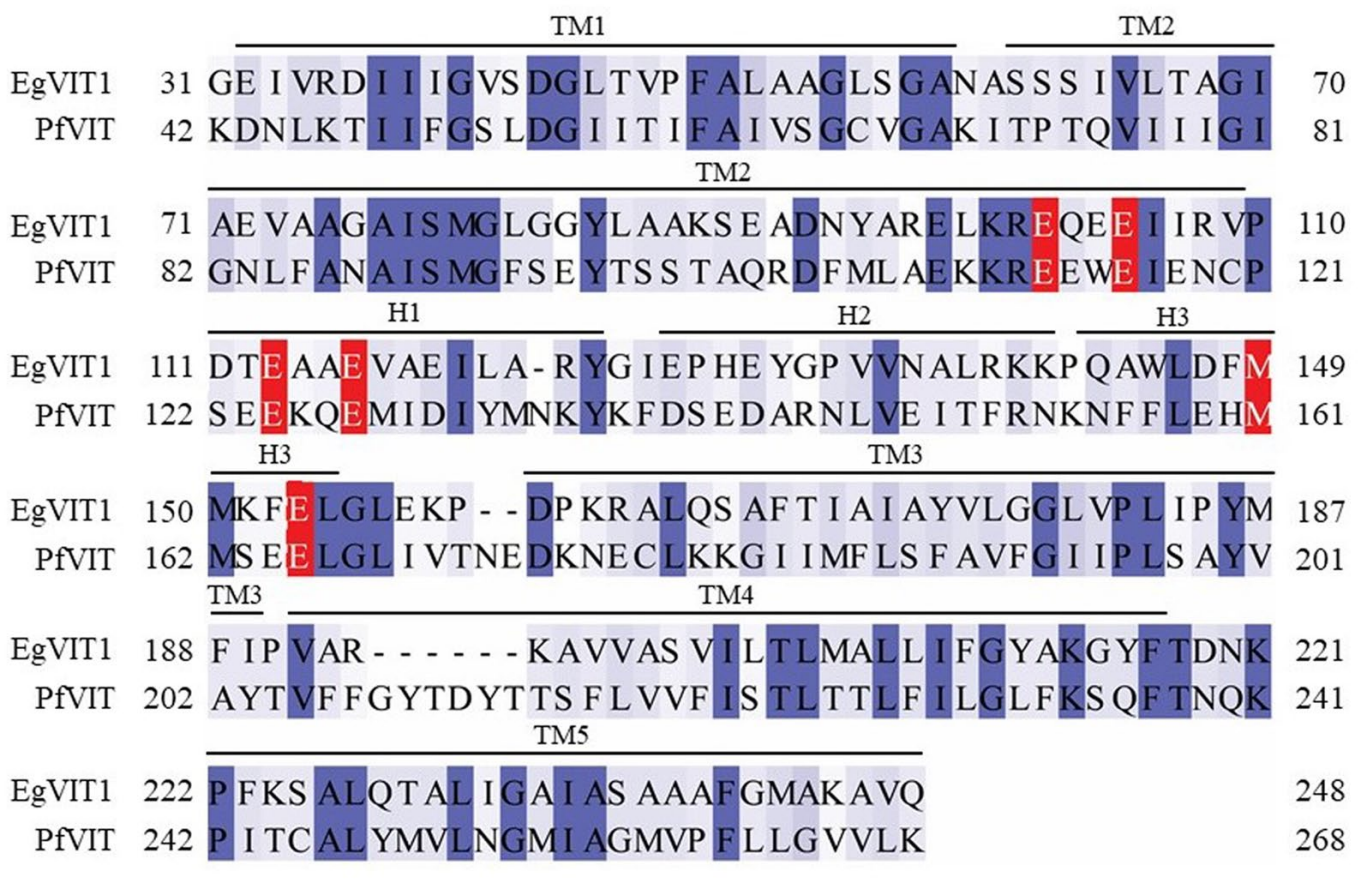
Primary sequence alignment of the modelled residues of PfVIT with the plant *Eg*VIT1 template. The target-template alignment used for construction of the PfVIT homology model was generated by HHblits [49]. Conserved, similar, and non-conserved amino acids are highlighted in dark blue, light blue, and white, respectively. Conserved glutamate and methionine residues involved in coordination of substrate metal cation(s) within the cytoplasmic metal binding domain (MBD) are presented in red boxes. The positions of the transmembrane spanning helices (TM1 to TM5) and helices H1 to H3 of the cytoplasmic MBD of the plant VIT1 structure are indicated

The need for a more complete understanding of the mechanisms of metal ion recognition and transport by the *P. falciparum* vacuolar iron transporter PfVIT encouraged the production of a comparative structural model of the protein to act as a guide for experiments aimed at deciphering its metal substrate binding site(s). The best-quality structural model of PfVIT was built using the automated comparative modelling web-server SWISS-MODEL. The backbone coordinates for the final homology model were built based on the 3.5 Å crystal structure of *Eucalyptus grandis* vacuolar iron transporter VIT1 (PDB ID: 6IU4) [31]. Each protomer of the model consisted of 227 residues (42–268) of PfVIT which were aligned with residues 31–248 of the VIT1 template sequence (Fig. 1). There was 29% sequence identity and 34% sequence similarity over this range. The model excluded N-terminal residues 1–41 and C-terminal residues 269–273 of PfVIT. The overall average G-factor of the model was − 0.03 and it had no unusual stereochemical properties. Ramachandran plot analysis revealed 97.1% and 2.9% of non-glycine and non-proline residues were located in the favoured and additional allowed regions, respectively; no residues were found in the generously allowed and disallowed regions (Additional file 3: Figure S2). These data are all indicative of a good-quality model.

**Fig. 2:**
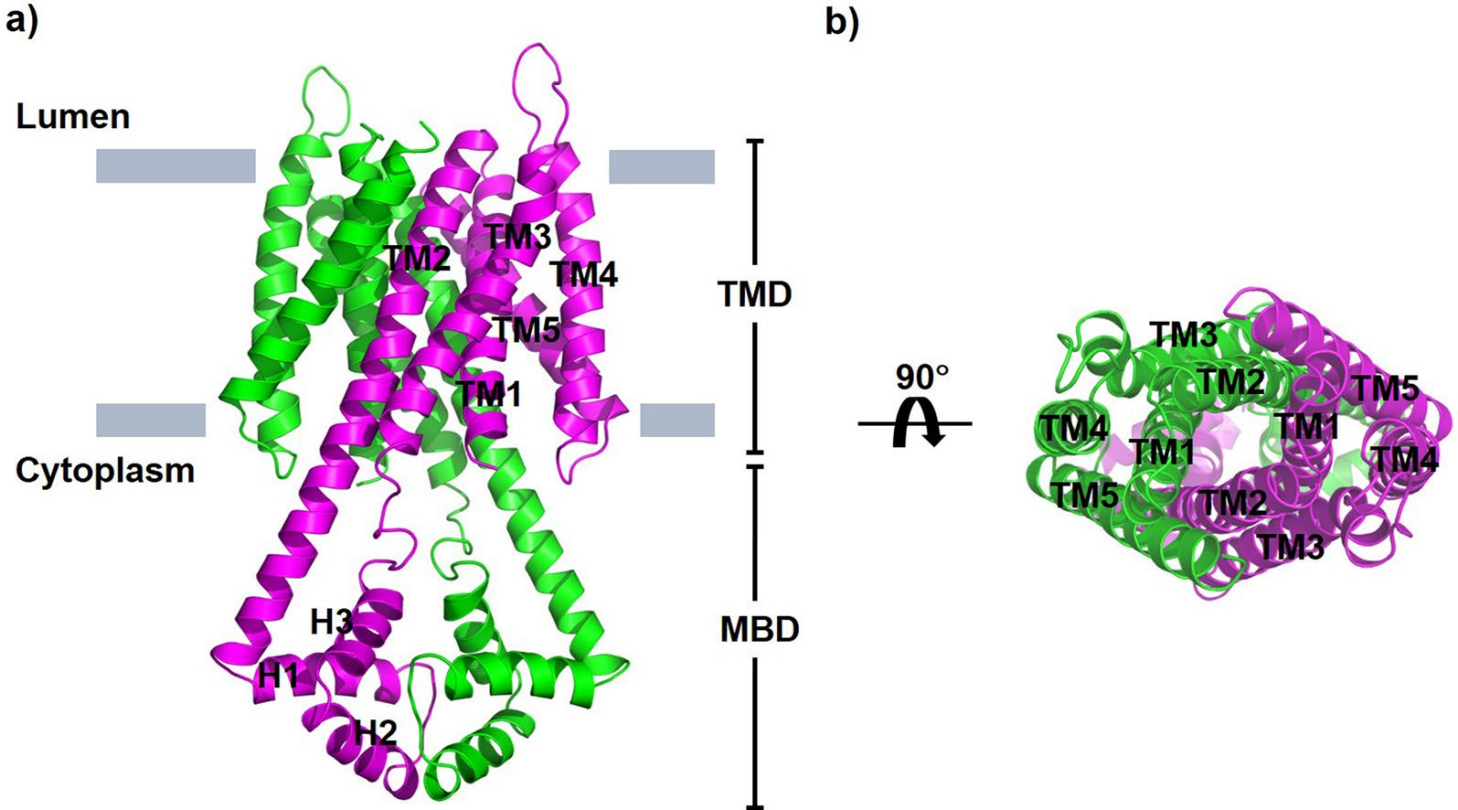
Structural model of the *P. falciparum* vacuolar iron transporter PfVIT. **a** The PfVIT structural model as viewed parallel to the plane of the interior compartment membrane, the boundaries of which are represented by grey rectangles. The protomers of the homodimeric structure are represented as ribbon structures and coloured magenta and green. The transmembrane domain (TMD) and cytoplasmic metal binding domain (MBD) are indicated and the individual helices of each are labelled TM1-TM5 and H1-H3, respectively, on one of the protomers. The N-and C-termini of the protein are located on the cytoplasmic and lumenal sides of the membrane, respectively. **b** Arrangement of the transmembrane spanning helices of the PfVIT structural model viewed from the lumenal side of the membrane

The biologically relevant structure of VIT1 is a homodimer [31] and it is probable that PfVIT also functions as such. Not unexpectedly, the overall fold of the PfVIT model is very similar to that of its plant VIT1 homologue with each subunit of the homodimer composed of two domains: a transmembrane domain (TMD) and a cytoplasmic metal binding domain (MBD) (Fig. 2). Each PfVIT protomer consists of a bundle of five transmembrane (TM) helices in a 2 + 3 arrangement typical of CCC1/VIT1 family members [31], and with the N- and C-termini located on the putative cytoplasmic and compartmental lumen sides of the membrane, respectively (Fig. 2a). Viewed perpendicular to the plane of the membrane from the lumenal side of the protein, TMs 2–5 of each protomer are arranged in counter-clockwise sequence around the central TM1, and TM5 is tilted by about 30° to the membrane normal (Fig. 2b). TM2 extends into the cytoplasm and connects to three short helices (H1-H3). Dimerization of PfVIT in the model occurs via interactions between both the TMD and MBD domains of each protomer. At the dimer interface of the TMD, extensive non-bonding and some H-bonding interactions between TM1, TM2 and TM5 of each protomer are the main contributors to the dimeric interaction. In the MBD, in addition to H-bonding and hydrophobic interactions, the model suggests that salt bridges between the protomers also contribute to dimerization. A large, funnel-shaped cavity which is accessible to the cytoplasmic solvent is located at the dimer interface between the TMDs. This cavity is lined with acidic, basic and sulphur-containing residues from each protomer, and it is likely that these form a hydrophilic translocation pathway across the membrane for the metal ion substrate. The alternating access model of membrane transport, however, dictates that the substrate binding site of any transporter cannot be accessible simultaneously to both sides of the membrane [39]. Further inspection of the PfVIT model revealed the luminal side of the protein to be sealed by a ‘plug’ of hydrophobic residues located on TM1 and TM2 of the protein (Additional file 4: Figure S3). Therefore, it is likely the model is representative of PfVIT in an ‘inward-open’ conformational state with the luminal side of the protein closed to the iron storage compartment interior.

**Fig. 3:**
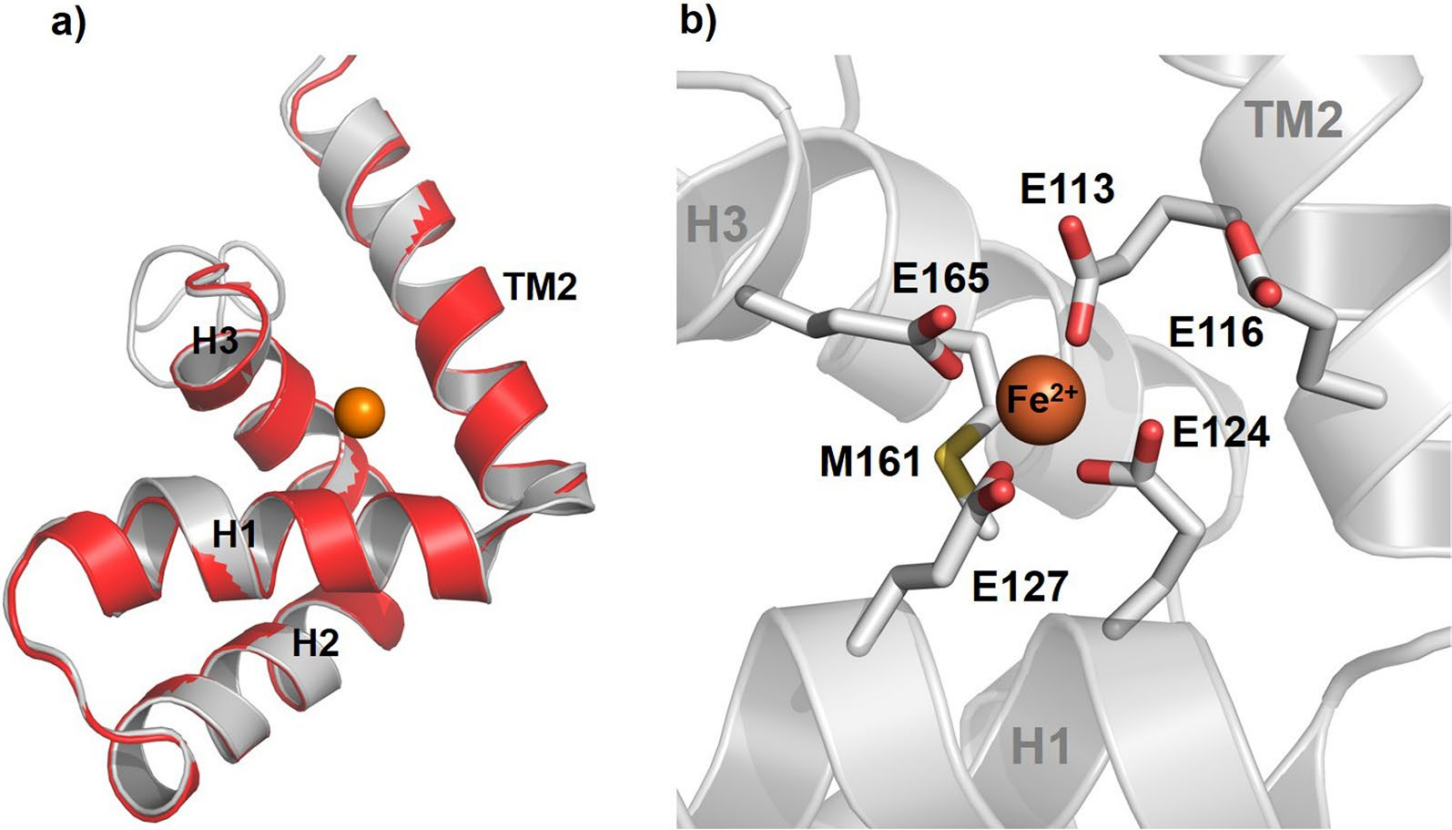
Structural model of the PfVIT cytoplasmic metal binding domain. **a** Structural alignment of the PfVIT cytoplasmic MBD structural model (coloured grey) with the crystal structure (PDB ID: 6IU9) [31] of plant VIT1 MBD (coloured red). Bound ferrous cation is represented as an orange sphere. **b** Structural model of the PfVIT MBD substrate binding site. The side chains of the amino acid residues that form the putative binding site are represented as sticks with oxygen atoms coloured red and sulphur atoms yellow. The coordinated Fe^2+^ cation is represented as an orange sphere

In the plant VIT1 structure, side chains of five highly conserved glutamate residues (E102, E105, E113, E116 and E153) and a single conserved methionine residue (M149) that are located on the cytoplasmic end of TM2, and on helices H1 and H3 of the MBD form a metal binding site [31]. Comparison of our PfVIT model with the VIT1 structure, in combination with sequence and structural alignments, suggested negatively charged amino acid residues E113 and E116 on TM2, E124 and E127 on H1, and M161 and E165 on H3 as the equivalent binding site residues in PfVIT. To investigate possible interactions between these residues and bound Fe^2+^ the structure of isolated MBD was modelled using the 3.0 Å crystal structure of the MBD fragment of plant VIT1 bound to Fe^2+^ as the template (PDB: 6IU9) [31]. The initial best model of PfVIT MBD, which consisted of 79 residues (A101-L179), was further optimised, refined and energy minimised to produce a structure with 92% and 5.3% of residues located in the most favoured and additionally allowed regions, respectively, of a Ramachandran plot (Additional file 3: Figure S2). One residue (E73) resided in the generously allowed region of the plot and a further residue (V70) in the disallowed region. The latter is not surprising because V70 is located on H3 at the C-terminal end of the PfVIT MBD and this part of the helix was unstructured in the isolated plant VIT MBD crystal structure. Superposition of the PfVIT MBD homology model with the VIT1 MBD crystal structure revealed excellent structural alignment (Fig. 3a). The atomic coordinates of the bound Fe^2+^ ion from the superposed VIT1 MBD structure were extracted and transferred to the PfVIT MBD homology model to yield a final model of PfVIT cytoplasmic MBD with a single bound Fe^2+^ (Fig. 3b) and this enabled a detailed structural analysis of the interactions between metal ion substrate and individual amino acid residues. Analysis by the CheckMyMetal tool [36] suggested substrate Fe^2+^ to be octahedrally coordinated by oxygen atoms of E113, E124, E127 and E165 and the sulphur atom of M161 (Table 1). In the model the carboxylic oxygens of the E116 side chain were both positioned too distant (4.4 Å) from the Fe^2+^ ion to be involved directly in its coordination. The PfVIT homology model was subsequently used to guide mutagenesis studies designed to determine the individual amino acid residues essential for coordination of Fe^2+^ to the PfVIT metal binding domain and, therefore, to transporter function.

**Table 1:** Distances between putative PfVIT cytoplasmic MBD substrate-binding residues and Fe^2+^ substrate ion

Residue	Coordinating atom	Distance to Fe^2+^
E113	Oε2	1.78 Å
E124	Oε1	1.99 Å
E127	Oε1	2.21 Å
M161	Sδ	2.99 Å
E165	Oε1	1.76 Å
E165	Oε2	3.30 Å

### The recombinant sPfVIT construct is localized to the yeast vacuole membrane

**Fig. 4:**
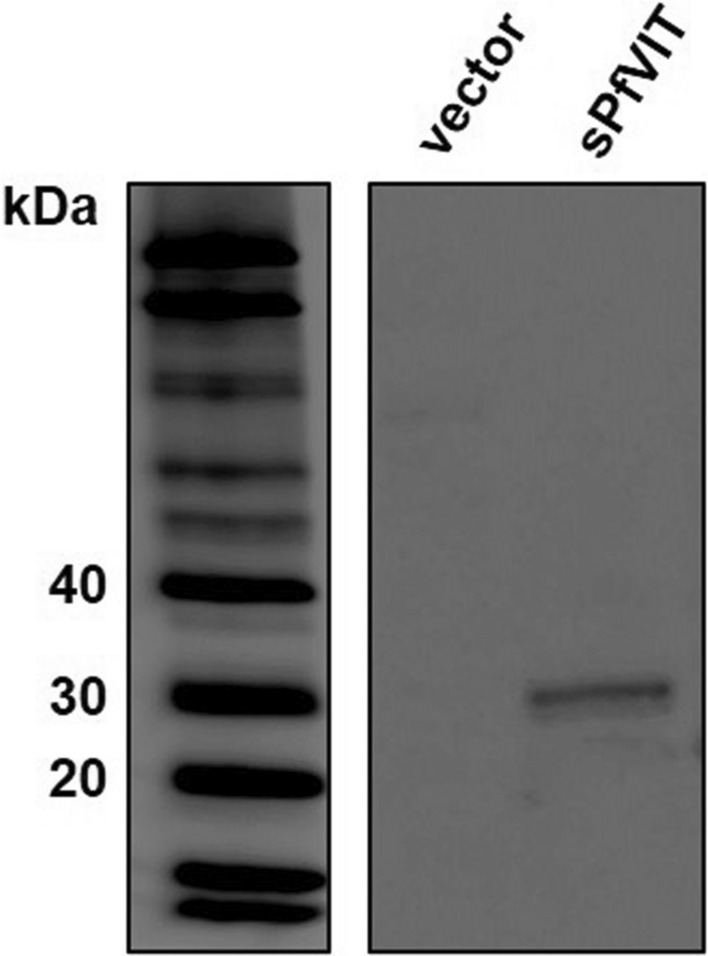
Western blot of vacuolar preparations from *ccc1Δ S. cerevisiae* cells. 7 µg of protein from vacuoles prepared from *ccc1Δ* yeast cells that either harboured ‘empty’ pESC-Leu (vector) or overexpressed wild type sPfVIT transporter was loaded onto each lane of the gel. The left-hand panel shows the molecular weight markers. Localization of heterologously overexpressed sPfVIT to the yeast vacuole was confirmed by detection of the His-tag of the recombinant protein

Prior to performing a functional assessment of the malaria transporter in the yeast model system it was necessary to demonstrate localization of the sPfVIT construct used in the study. Although previously published work showed that N-terminally truncated sPfVIT was targeted correctly to the yeast vacuole, the construct used in that study did not possess any C-terminal histidine affinity tag [21]. Therefore, to ensure that the presence of the C-terminal decahistidine tag on the recombinant transporter used for the current study did not confound correct targeting to the yeast vacuole membrane, western blot analysis of isolated vacuoles prepared from *ccc1Δ* yeast cells that overexpressed the protein from pESC-Leu-sPfVIT plasmid was performed. Vacuoles prepared from cells that harboured empty vector were used as a negative control. As shown in Fig. 4, the western blot of the isolated vacuolar proteins prepared from cells that overexpressed the transporter revealed a single band with apparent molecular mass of ~ 30 kDa that corresponded to the sPfVIT construct, thereby demonstrating correct localization of the protein to the yeast vacuole.

### Glutamate residues within the cytoplasmic metal binding domain of PfVIT are replaceable

To assess the functional role of individual amino acid residues in formation of the putative Fe^2+^ binding site of PfVIT, site directed mutagenesis was performed and the ability of mutant protein to rescue an iron-sensitive yeast phenotype from the toxic effects of high extracellular iron concentrations was tested in a complementation assay [21, 31]. For this assay, the *ccc1∆* strain of *S. cerevisiae* that overexpressed transporter mutants from pESC-Leu-sPfVIT plasmid (see Section “Methods”) was spotted onto solid media plates that contained either no added iron or 7.5 mM iron added in the form of ammonium iron (II) sulphate. Cells that expressed wild type sPfVIT or harboured ‘empty’ pESC-Leu vector were used as positive and negative controls, respectively.

**Fig. 5:**
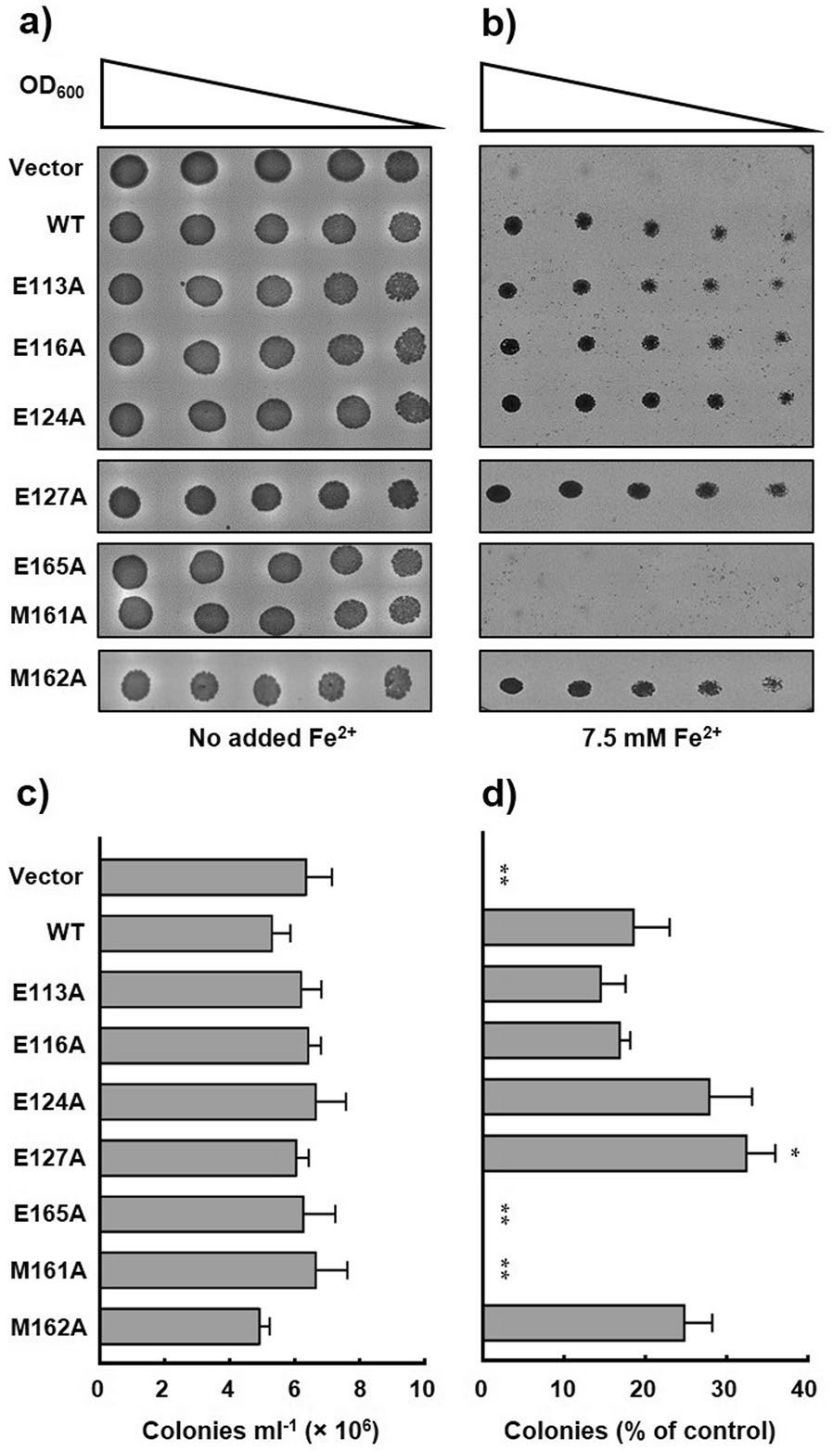
Iron tolerance of *ccc1Δ* yeast conferred by the functional expression of PfVIT cytoplasmic metal binding domain alanine mutants. Cultures of *ccc1Δ* yeast cells that expressed wild type sPfVIT, alanine mutant sPfVIT or empty pESC-Leu vector were diluted in twofold steps (from OD_600_ 0.2 to 0.0125), spotted onto SC agar plates lacking leucine and supplemented with **a** no added Fe^2+^ as a control or **b** 7.5 mM Fe^2+^. The plates were incubated at 30 °C for 3 days. Fe^2+^ was provided as ammonium FeSO_4_ in the presence of 2 mM ascorbic acid. **c** Quantitative analysis of colony growth (counted as colonies formed per ml of OD1 culture) of *ccc1Δ* yeast cells that expressed wild type sPfVIT, alanine mutant sPfVIT or empty pESC-Leu vector on SC-Leu agar plates that contained no added iron. **d** Colony formation of *ccc1Δ* yeast cells that expressed wild type sPfVIT, alanine mutant sPfVIT or empty pESC-Leu vector on SC-Leu agar plates that contained 7.5 mM Fe^2+^. For each yeast transformant, colony formation is quantitated as a percentage of that of the respective control grown on plates with no added iron. Data in **c** and **d** are presented as the mean ± s.e.m. of three independent experiments. The data were analysed using one-way analysis of variance (ANOVA) and Dunnett’s multiple comparison tests to determine any statistically significant difference between growth of yeast cells that expressed wild type and mutant sPfVIT; *P < 0.05, **P < 0.01

Initially, an alanine scan of the putative binding site glutamate (E113, E116, E124, E127 and E165) and methionine (M161) residues was performed to investigate the necessity of those residues for transporter function. Although the structural model of the MBD did not indicate a direct role for the methionine at position 162 in binding of Fe^2+^, given its proximity to the putative metal binding site the effect of mutation of M162 on transporter function was also tested. Qualitative and quantitative analyses demonstrated that overexpression of mutant transporter resulted in no statistically significant differences in yeast cell growth in the absence of externally added iron (Fig. 5a, c), thereby confirming no detrimental effect on yeast cell growth due to overexpression of each transporter. However, on media that contained added iron, negative control yeast cells that harboured ‘empty’ pESC-Leu vector were incapable of growth due to lack of a transporter that could function in vacuolar sequestration of excess cytoplasmic iron (Fig. 5b, d). Overexpression of recombinant wild type sPfVIT in *ccc1*∆ yeast, however, successfully rescued the iron-sensitive phenotype indicating that our N-terminally truncated PfVIT construct can function as the vacuolar iron transporter in *S. cerevisiae*. Overexpression of E113A, E116A, E124A, E127A and M162A mutants of sPfVIT also rescued the iron-sensitive yeast phenotype. Cells that overexpressed the E113A, E116A, E124A and M162A mutants grew similarly to cells that overexpressed wild type transporter, but quantitative analysis revealed E127A to be a mild gain of function mutation (Fig. 5d). Taken together, these data suggest a negative charge at amino acid positions 113, 116, 124 or 127 is therefore not a requirement for PfVIT-catalysed iron transport. In contrast, sPfVIT E165A and M161A were loss of function mutants that failed to complement the growth inhibition of *ccc1*Δ yeast on media containing added iron (Fig. 5b, d), suggesting an important functional role for E165 and M161 in recognition and/or binding of Fe^2+^ substrate in the malaria transporter.

**Fig. 6:**
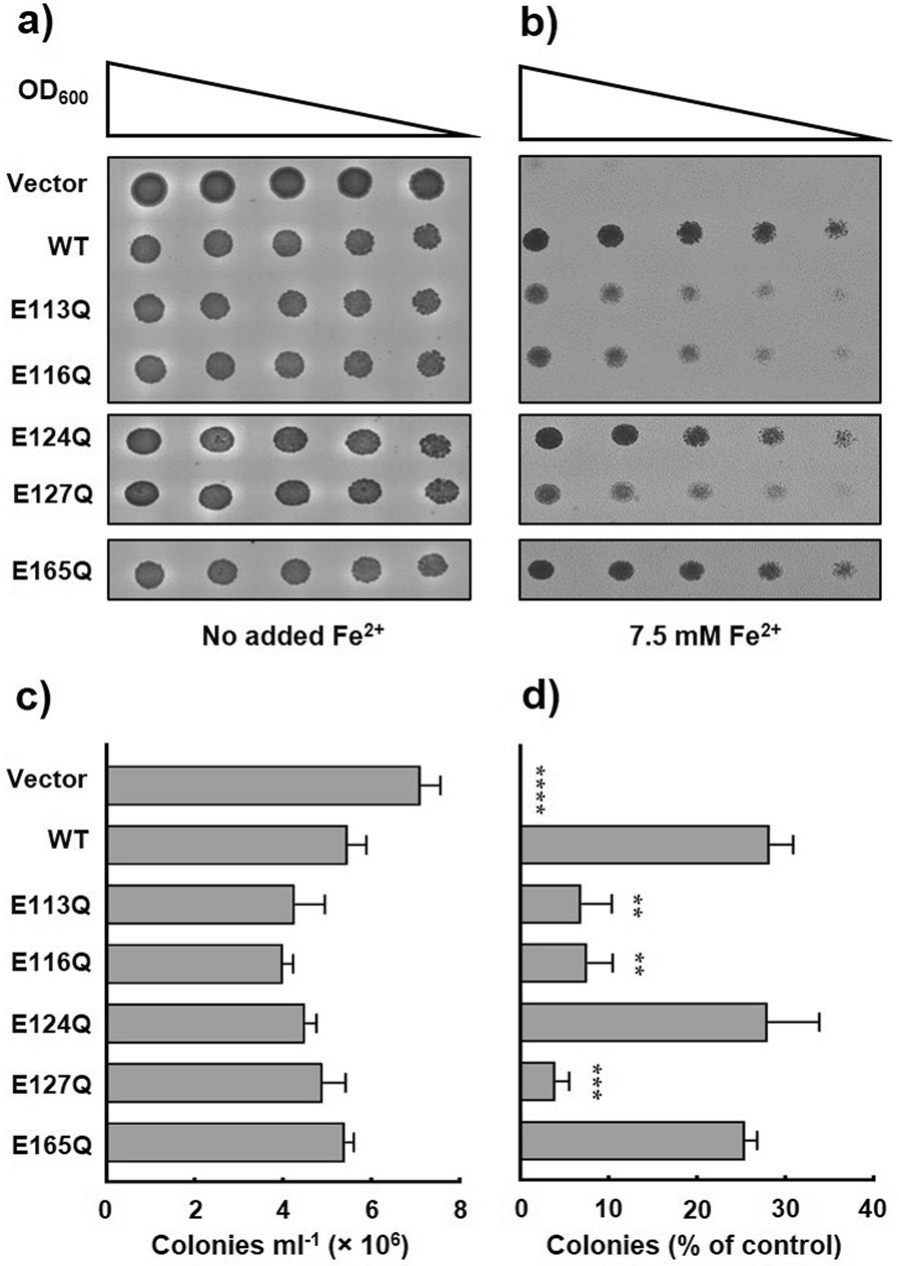
Iron tolerance of *ccc1Δ* yeast conferred by the functional expression of PfVIT cytoplasmic metal binding domain glutamine mutants. Cultures of *ccc1Δ* yeast cells that expressed wild type sPfVIT, glutamine mutant sPfVIT or empty pESC-Leu vector were diluted in twofold steps (from OD_600_ 0.2 to 0.0125), spotted onto SC agar plates lacking leucine and supplemented with **a** no added Fe^2+^ as a control or **b** 7.5 mM Fe^2+^. The plates were incubated at 30 °C for 3 days. **c** Quantitative analysis of colony growth (counted as colonies formed per ml of OD1 culture) of *ccc1Δ* yeast cells that expressed wild type sPfVIT, glutamine mutant sPfVIT or empty pESC-Leu vector on SC-Leu agar plates that contained no added iron. **d** Colony formation of *ccc1Δ* yeast cells that expressed wild type sPfVIT, glutamine mutant sPfVIT or empty pESC-Leu vector on SC-Leu agar plates that contained 7.5 mM Fe^2+^. For each yeast transformant, colony formation is quantitated as a percentage of that of the respective control grown on plates with no added iron. Data in **c** and **d** are presented as the mean ± s.e.m. of three independent experiments. The data were analysed using one-way analysis of variance (ANOVA) and Dunnett’s multiple comparison tests to determine any statistically significant difference between growth of yeast cells that expressed wild type and mutant sPfVIT; **P < 0.01, ***P < 0.001, ****P < 0.0001

Individual replacement of the negatively charged glutamate residues that form the putative binding site with neutral glutamine at positions 113, 116 and 127 severely impaired complementation efficiency of the iron-sensitive *ccc1*∆ phenotype in the yeast complementation assays (Fig. 6). As shown in Fig. 6b, d, yeast cells that overexpressed the E124Q and E165Q sPfVIT mutants exhibited complementation efficiency that was statistically indistinguishable from that of cells that overexpressed the wild type transporter. Furthermore, the data suggest that position 124 of the MBD is particularly resilient to amino acid substitution.

### Methionine at position 161 is crucial for PfVIT function

**Fig. 7:**
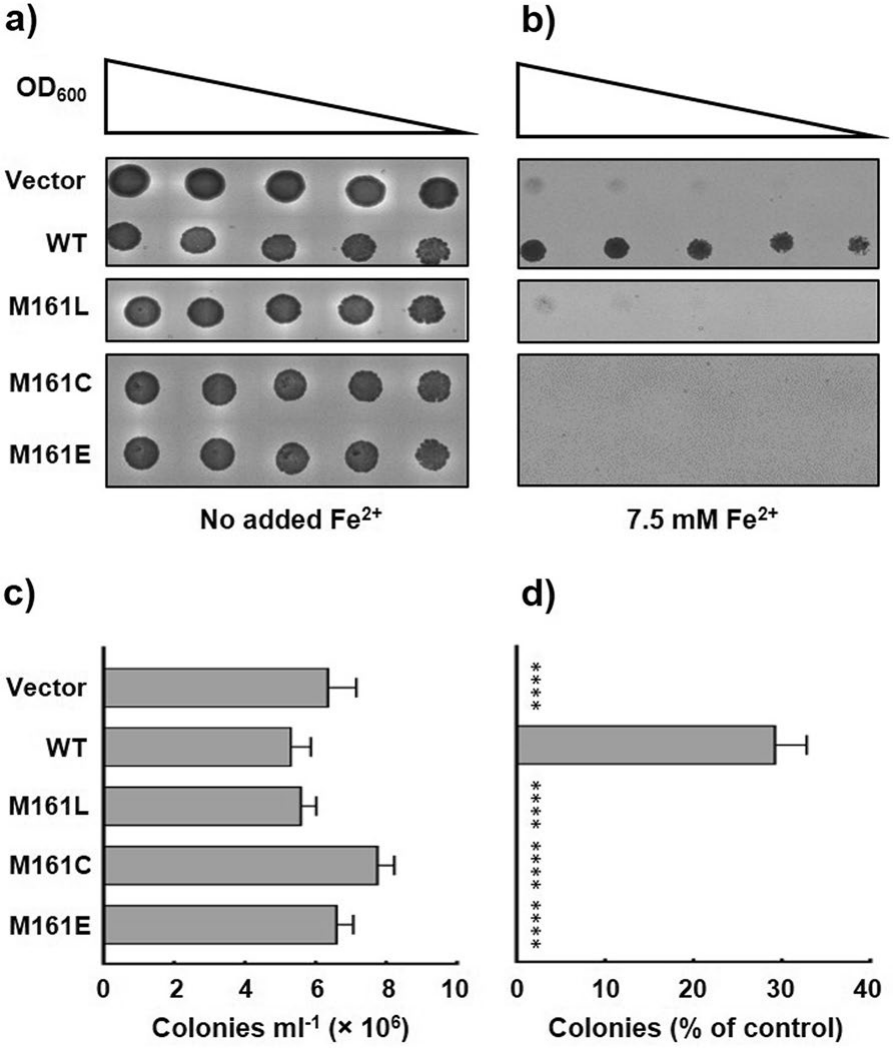
Effect of substitution of the PfVIT cytoplasmic metal binding domain methionine 161 residue on iron tolerance of *ccc1Δ* yeast. Cultures of *ccc1Δ* yeast cells that expressed wild type sPfVIT, methionine 161 mutant sPfVIT or empty pESC-Leu vector were diluted in twofold steps (from OD_600_ 0.2 to 0.0125), spotted onto SC agar plates lacking leucine and supplemented with **a** no added Fe^2+^ as a control or **b** 7.5 mM Fe^2+^. The plates were incubated at 30 °C for 3 days. **c** Quantitative analysis of colony growth (counted as colonies formed per ml of OD1 culture) of *ccc1Δ* yeast cells that expressed wild type sPfVIT, methionine mutant sPfVIT or empty pESC-Leu vector on SC-Leu agar plates that contained no added iron. **d** Colony formation of *ccc1Δ* yeast cells that expressed wild type sPfVIT, methionine mutant sPfVIT or empty pESC-Leu vector on SC-Leu agar plates that contained 7.5 mM Fe^2+^. For each yeast transformant, colony formation is quantitated as a percentage of that of the respective control grown on plates with no added iron. Data in **c** and **d** are presented as the mean ± s.e.m. of three independent experiments. The data were analysed using one-way analysis of variance (ANOVA) and Dunnett’s multiple comparison tests to determine any statistically significant difference between growth of yeast cells that expressed wild type and mutant sPfVIT; ****P < 0.0001

Alanine scanning mutagenesis suggested a methionine residue at position 161 in the MBD was required for transporter function (Fig. 5). To investigate further if M161 is an essential residue in PfVIT, additional mutagenic analysis was performed. The methionine was substituted with leucine (M161L) and cysteine (M161C) to test the functional significance of amino acid side chain bulk and the presence of a sulphur atom, respectively, at position 161. An M161E mutant was constructed to test if a negative charge could compensate for loss of the methionine. As shown in Fig. 7, replacement of the methionine with leucine, cysteine or glutamate completely abolished the ability of PfVIT to rescue the iron-sensitive *ccc1*∆ yeast phenotype. Therefore, M161 is an essential residue for PfVIT function.

**Fig. 8:**
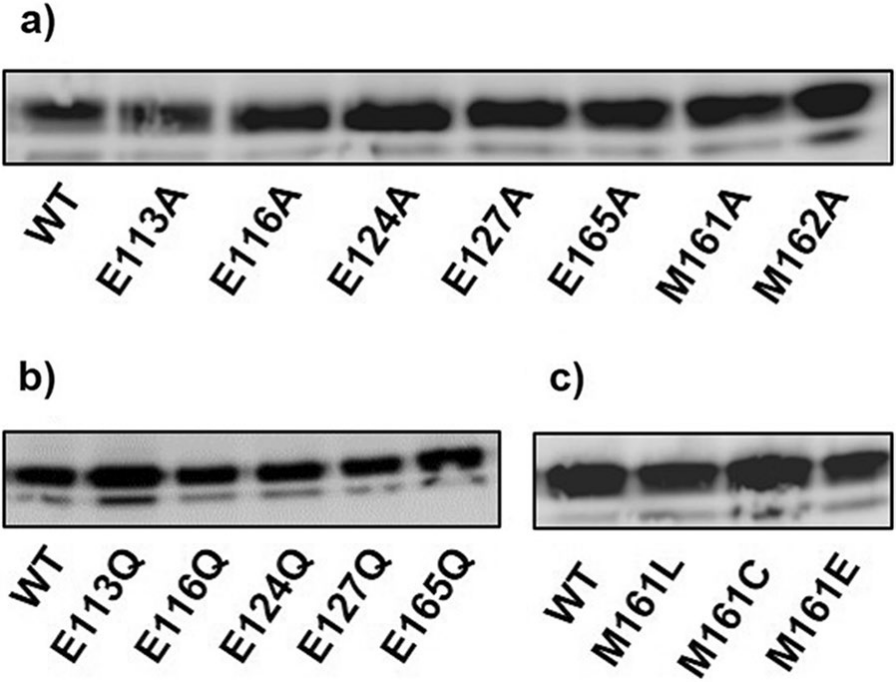
Western blot analysis of expression levels of wild type and mutant sPfVIT in *ccc1Δ* yeast cells. Expression levels of **a** wild type (WT) sPfVIT and alanine mutant sPfVIT; **b** wild type (WT) sPfVIT and glutamine mutants sPfVIT, and **c** wild type (WT) sPfVIT and M161 leucine, cysteine and glutamate mutants of the transporter

Western blot analysis of yeast total protein confirmed that expression levels of the N-terminal truncation wild type and mutant PfVIT were similar (Fig. 8). Therefore, measured differences in complementation efficiency of the iron-sensitive *ccc1*∆ phenotype in the assays were due solely to the effect(s) of amino acid substitution and not to differences in expression levels of the recombinant transporter mutants.

## Discussion

Despite the gradual annual decline in malaria cases and deaths over the past decade, emerging and growing resistance to frontline drugs used to treat the disease has highlighted a need for identification and characterisation of novel anti-malarial drug targets [25, 40]. A vacuolar iron transporter family homologue that functions in iron detoxification during liver and asexual blood stages of *Plasmodium* species could represent just such a target for novel prophylactic drugs [21, 41]. In the absence of an experimentally determined three-dimensional structure of any *Plasmodium* VIT, a high-quality homology model of PfVIT from the human malaria parasite *P. falciparum* was built and used as a guide for mutagenesis studies aimed at identification of individual amino acid residues that constitute the substrate binding site of the protein; information critical not only for a better understanding of PfVIT function but also for structure-based design of novel inhibitors of the transporter.

The structural similarities between the PfVIT homology model and plant VIT1 crystal structure suggest a common gross architecture for eukaryotic VITs. This similarity is particularly evident in the cytoplasmic MBD of the transporters; several glutamate and methionine residues that form the metal binding site(s) of plant VIT1 are not only fully conserved in the primary sequence of PfVIT, but the binding sites also exhibit a striking degree of structural conservation. Despite this, the malaria and plant VITs possess different metal cation substrate recognition profiles. PfVIT is specific for Fe^2+^ whereas plant VITs are more promiscuous and can bind and transport divalent Zn, Mn, Co or Ni cations in addition to Fe^2+^ [12, 21, 27, 31]. Yeast CCC1 is also capable of transporting Mn^2+^ and Ca^2+^ ions [10]. Differences between *Plasmodium* and plant or yeast VITs may also extend to metal ion occupancy of the cytoplasmic MBD. Although our PfVIT model suggested only one Fe^2+^ ion is bound to the MBD, the crystal structure of plant VIT1 revealed that two Zn^2+^ ions and a water molecule played a role in coordination of Fe^2+^ to that transporter [31]. While it is intriguing to speculate if PfVIT can bind more than one metal ion in the MBD, or if Zn^2+^ is a cofactor necessary for PfVIT function, previous work demonstrated that purified recombinant PfVIT in detergent solution did not bind exogenously added zinc [27].

The results of yeast complementation assays that assessed the ability of mutant PfVIT to rescue the *ccc1*Δ iron sensitive phenotype of *S. cerevisiae* suggested a plasticity of the metal binding site with respect to substrate binding and highlighted additional differences between the *P. falciparum* and plant VITs. Taken together, the assay results clearly demonstrated that a glutamate residue at positions 113, 116, 124, 127 or 165 in the PfVIT metal binding domain is not essential for transporter function. Individual removal of the negative charge at each of these positions by alanine scanning mutagenesis revealed only E165 to be sensitive to replacement by alanine. However, introduction of a neutral glutamine at the same position completely recovered the iron-tolerant yeast phenotype. Furthermore, substitution of glutamate by glutamine at positions 113, 116 and 127 greatly diminished but did not completely abolish the ability of *ccc1*Δ yeast cells that expressed the mutant transporters to grow on medium that contained added iron. Introduction of a glutamine at position 124 had no significant effect on PfVIT function. In contrast, loss of the negative charge by individual replacement of the equivalent MBD glutamate residues with glutamine in plant VIT1 resulted in loss of function of all the mutant transporters [31]. The resilience exhibited by PfVIT toward substitution of the conserved MBD glutamate residues suggests the malaria protein can remodel its binding site to utilise different coordination geometries for binding the Fe^2+^ substrate in response to mutation. This contention is supported by a study of transition metal binding selectivity in proteins which showed that although Fe^2+^ had a preference for octahedral coordination geometry, other Fe^2+^-binding geometries with coordination numbers of 4 or 5 were also well represented in the set of test proteins [42]. It is also possible that water molecules in OH^−^ or O^2−^ form could replace individual amino acid side chains as coordinating ligands to bind metal substrate in the mutant PfVIT proteins, as observed in the plant VIT1 structure [31]. Although the homology model of PfVIT suggested a single Fe^2+^ was bound to each MBD of the protein, the possibility of the presence of more than one distinct Fe^2+^ binding site within the domain cannot be excluded; clarification of this will likely have to await the availability of an experimentally-determined, high resolution structure of PfVIT with substrate bound.

Regardless of the limitations of the PfVIT homology model, it can still provide a useful framework for interpretation of the mutagenesis experiments. The statistically significant gain of function observed for the PfVIT E127A mutation can be rationalised if consideration is given to the location of this residue on the cytoplasmic MBD of the protein. Inspection of the PfVIT MBD structure (Fig. 3b) revealed the acidic E127 to be located about midway on H1 of the MBD and facing toward the cytosol. Such positioning could allow E127 to function as a ‘gatekeeper residue’ to control entry/exit of Fe^2+^ to/from the metal binding site. It is plausible, therefore, that the gain of function observed for the E127A mutant was due to removal of side chain bulk. Furthermore, it suggests that the negative charge on E127 may play an important role in controlling the binding of Fe^2+^ to the MBD. Consistent with the notion of E127 as a gatekeeper residue was the loss of function observed when the acidic, negatively charged glutamate at this position was substituted with uncharged glutamine.

The information provided by the PfVIT homology model also allowed the effects of mutation of the E165 residue to be placed in a structural context. The loss of function observed when E165 was substituted with alanine, combined with the ability of the PfVIT E165Q mutant to rescue the iron-sensitive of *ccc1*Δ yeast phenotype, suggested an important role for side chain bulk at position 165 for transporter function. The location of E165 at the C-terminal end of helix 3 of the MBD (see Fig. 3b), and with its side chain facing towards the substrate translocation pore of PfVIT, places this glutamate residue at an ideal position to facilitate diffusion of Fe^2+^ along the protein surface from the binding site to the central pore of the transporter. PfVIT functions as an Fe^2+^/H^+^ exchanger [27] and competition between protons and substrate is central to the transport mechanism of these proteins [43]. The apparent necessity of an amino acid side chain that is capable of undergoing (de)protonation at position 165 of PfVIT raises the possibility that E165 could be a component of a proton relay that functions in translocation of H^+^ countersubstrate across the internal compartment membrane. Further biochemical studies aimed at measurement of substrate binding to purified wild type and mutant transporter in detergent solution combined with transport measurements of protein reconstituted into liposomes will probably be required to confirm if this is indeed the case.

In contrast to the conserved glutamate residues of the PfVIT cytoplasmic MBD, a methionine at position 161 is indispensable to transporter function. Sulphur-containing residues play an important role in coordination of metal ions in the binding sites of many metalloproteins [44, 45], and a conserved methionine in the metal binding site of plant VIT1 [31] and the Nramp-family of transporters [46] acts to confer metal ion selectivity for transition metal ions while discriminating against the alkaline earth metals. It is probable, therefore, that M161 performs the same selectivity function in PfVIT. Insight into the role of this methionine in metal ion selectivity can be provided by viewing substrate binding through the lens of the Lewis concept of acids and bases [47]. Transition metal ions such as Fe^2+^ are regarded by Lewis theory as borderline acids with properties that are intermediate to those of the electrophilic hard Lewis acids and the nucleophilic soft Lewis acids [48]. The intermediate acid nature of Fe^2+^ means it can bind to both hard and soft base ligands such as the carboxylate oxygens of glutamate side chains, and the thioether sulphur atom of methionine side chains, respectively. The methionine side chain, however, is rather hydrophobic and an unsuitable ligand for the hard acid alkaline earth metals. Although this characteristic imparts a discriminatory function to methionine with respect to metal ion recognition, it does not fully explain why plant VIT1 can recognize and transport Co^2+^, Ni^2+^ and Zn^2+^ as well as Fe^2+^ [31] whereas PfVIT is selective for Fe^2+^ [21, 27]. Therefore, other subtle specificity determinants must be at play within the PfVIT binding site to enable discrimination between Fe^2+^ and the other divalent transition metal cations. It may be that specific physicochemical attributes of Fe^2+^, such as size and electron configuration, are exploited by the transporter to sculpt the composition and geometry of the metal binding site and the immediate surroundings to afford selectivity.

## Conclusion

This work represents the first systematic characterization of the metal binding site of a non-plant VIT. Although the homology model of PfVIT provided a useful and effective guide for biochemical investigations of transporter function there are still several questions about metal ion binding to PfVIT that are outstanding and that cannot be answered by homology modelling alone. These include: (i) Can more than one Fe^2+^ bind to the MBD? (ii) Are divalent metal ions other than iron substrates of the MBD? (iii) Can other metals bind to PfVIT MBD simultaneously with iron? (iv) If so, are those metals necessary cofactors for PfVIT function? It is also unknown if the integrity of the metal binding site is retained or if different residues become involved in substrate binding as the transporter undergoes the conformational changes typically associated with active transport processes. Nevertheless, the availability of a high-quality homology model of PfVIT offers the possibility of further computational studies of iron binding to the protein by molecular dynamics simulations to enable identification of other residues within the protein that are essential for function and opens the door for rational design of therapeutics to interfere with iron homeostasis within the malaria parasite.

## Supplementary Information

**Additional file 1: Figure S1.** Nucleotide sequence of the codon optimized PfVIT N-terminal truncation construct used in this study.

**Additional file 2: Table S1**. DNA oligonucleotide primers used in the study.

**Additional file 3: Figure S2.** Ramachandran plots of backbone dihedral angles for the three-dimensional structural models of PfVIT.

**Additional file 4: Figure S3.** Structural model of the functional PfVIT homodimer in an ‘inward open’ conformation and with Fe^2+^ substrate bound.

## Data Availability

The atomic coordinate (.pdb) files of the homology models described in this article are available from the corresponding author on request.

## Abbreviations

A: Alanine
BSA: Bovine serum albumin
C: Cysteine
CCC1: Cross complementer protein 1
E: Glutamic acid (glutamate)
*Eg*VIT1: *Eucalyptus grandis* vacuolar iron transporter 1
HRP: Horse radish peroxidase
L: Leucine
M: Methionine
MBD: Metal binding domain
PDB: Protein data bank
PfVIT: *Plasmodium falciparum* vacuolar iron transporter
Q: Glutamine
RT: Room temperature
sPfVIT: N-terminal truncation mutant of *Plasmodium falciparum* vacuolar iron transporter
SC-Leu: Single dropout formulation (without l-Leucine) of synthetic complete supplement mixture of amino acids
SDS-PAGE: Sodium dodecyl sulphate–polyacrylamide gel electrophoresis
TBS-T: Tris buffered saline-Tween buffer
TMD: Transmembrane domain
VIT: Vacuolar iron transporter
WT: Wild type
YPD: Yeast peptone dextrose medium
