# Supplementary material for: Characterization of the substrate binding site of an iron detoxifying membrane transporter from Plasmodium falciparum
Source: Malar J. 2021 Jun 30;20:295. doi: 10.1186/s12936-021-03827-7 (PMC8247066; doi:10.1186/s12936-021-03827-7)
Supplement: Supplementary file 1 — Additional file 1: Figure S1. Nucleotide sequence of the codon optimized PfVIT N-terminal truncation construct used in this study. [file 12936_2021_3827_MOESM1_ESM.pdf]

5’-ATGCATAATTTGGATAAGGATAATTTGAAGACTATCATCTTCGGTTCTTTAGATGGT
ATTATTACAATTTTTGCTATTGTTTCAGGTTGTGTTGGTGCAAAGATCACTCCAACAC
AAGTTATTATCATCGGTATCGGTAATTTGTTCGCTAACGCAATCTCTATGGGTTTTTC
AGAATACACTTCTTCAACAGCTCAAAGAGATTTCATGTTGGCAGAAAAGAAAAGAGA
AGAATGGGAAATCGAAAACTGTCCATCTGAAGAAAAGCAAGAAATGATCGATATCTA
TATGAATAAGTACAAGTTCGATTCAGAAGATGCTAGAAATTTGGTTGAAATCACTTTT
AGAAATAAGAATTTCTTTTTGGAACATATGATGTCTGAAGAATTGGGTTTAATCGTTA
CAAACGAAGATAAGAACGAATGTTTGAAGAAAGGTATTATTATGTTTTTATCTTTTGC
AGTTTTTGGTATTATTCCATTGTCAGCTTATGTTGCATACACTGTTTTCTTTGGTTACA
CAGATTACACTACATCATTTTTGGTTGTTTTTATTTCAACTTTGACTACATTGTTTATTT
TGGGTTTGTTTAAATCTCAATTCACTAACCAAAAGCCAATCACATGTGCTTTGTACAT
GGTTTTGAATGGTATGATTGCTGGTATGGTTCCATTTTTGTTAGGTGTTGTTTTGAAA
AATAACATCTCTGAATTAGTTCCAAGAGGTTCTTCAAAATTGGGTCCAGAACAAAAAT
TAATTTCAGAAGAAGATTTGAACTCTGCTGTTGACCACCACCATCACCATCATCACCA
TCACCATTGA-3’

Figure S1: Nucleotide sequence of the PfVIT N-terminal truncation construct used in this study. The construct was codon optimized for expression in *Saccharomyces cerevisiae*.
