# Supplementary material for: Characterization of the substrate binding site of an iron detoxifying membrane transporter from Plasmodium falciparum
Source: Malar J. 2021 Jun 30;20:295. doi: 10.1186/s12936-021-03827-7 (PMC8247066; doi:10.1186/s12936-021-03827-7)
Supplement: Supplementary file 2 — Additional file 2: Table S1. DNA oligonucleotide primers used in the study. [file 12936_2021_3827_MOESM2_ESM.pdf]

Table S1: DNA oligonucleotide primers used in the study.

Primer name	Oligonucleotide sequence (5'-3')	Primer length (mer)
E113A	**F**: ggacagttttcgatttcccattcagctcttttcttttctgccaacatg	48
	**R**: catgttggcagaaaagaaaagagctgaatgggaaatcgaaaactgtcc	48
E113Q	**F**: cagttttcgatttcccattcttgtcttttcttttctgccaacatg	45
	**R**: catgttggcagaaaagaaaagacaagaatgggaaatcgaaaactg	45
E116A	**F**: ttcttcagatggacagttttcgatagcccattcttctcttttcttttctg	50
	**R**: cagaaaagaaaagagaagaatgggctatcgaaaactgtccatctgaagaa	50
E116Q	**F**: cagatggacagttttcgatttgccattcttctcttttcttttc	43
	**R**: gaaaagaaaagagaagaatggcaaatcgaaaactgtccatctg	43
E124A	**F**: tatagatatcgatcatttcttgcttagcttcagatggacagttttcgatttc	52
	**R**: gaaatcgaaaactgtccatctgaagctaagcaagaaatgatcgatatctata	52
E124Q	**F**: gatatcgatcatttcttgcttttgttcagatggacagttttcgattt	47
	**R**: aaatcgaaaactgtccatctgaacaaaagcaagaaatgatcgatatc	47
E127A	**F**: gtacttattcatatagatatcgatcatagcttgcttttcttcagatggacagtttt	56
	**R**: aaaactgtccatctgaagaaaagcaagctatgatcgatatctatatgaataagtac	56
E127Q	**F**: attcatatagatatcgatcatttgttgcttttcttcagatggacagt	47
	**R**: actgtccatctgaagaaaagcaacaaatgatcgatatctatatgaat	47
E165A	**F**: cttcgtttgtaacgattaaacccaaagcttcagacatcatatgttccaaaaa	52
	**R**: tttttggaacatatgatgtctgaagctttgggtttaatcgttacaaacgaag	52
E165Q	**F**: gtttgtaacgattaaacccaattgttcagacatcatatgttccaaaa	47
	**R**: ttttggaacatatgatgtctgaacaattgggtttaatcgttacaaac	47
M161A	**F**: attaaacccaattcttcagacatagcatgttccaaaaagaaattcttatttctaaaagtgatttcaacca	70
	**R**: tggttgaaatcacttttagaaataagaatttctttttggaacatgctatgtctgaagaattgggtttaat	70
M161L	**F**: taaacccaattcttcagacatcaaatgttccaaaaagaaattcttatttctaaaagt	57
	**R**: acttttagaaataagaatttctttttggaacatttgatgtctgaagaattgggttta	57
M161C	**F**: attaaacccaattcttcagacatacaatgttccaaaaagaaattcttatttctaaaagtgatttcaacca	70
	**R**: tggttgaaatcacttttagaaataagaatttctttttggaacattgtatgtctgaagaattgggtttaat	70

M161E	**F**: attaaacccaattcttcagacatttcatgttccaaaaagaaattcttatttctaaaagtgatttcaacca	70
	**R**: tggttgaaatcacttttagaaataagaatttctttttggaacatgaaatgtctgaagaattgggtttaat	70
M162A	**F**: ttcgtttgtaacgattaaacccaattcttcagaagccatatgttccaaaaagaaattcttatttctaaa	69
	**R**: tttagaaataagaatttctttttggaacatatggcttctgaagaattgggtttaatcgttacaaacgaa	69
