# Supplementary material for: Characterization of the substrate binding site of an iron detoxifying membrane transporter from Plasmodium falciparum
Source: Malar J. 2021 Jun 30;20:295. doi: 10.1186/s12936-021-03827-7 (PMC8247066; doi:10.1186/s12936-021-03827-7)
Supplement: Supplementary file 3 — Additional file 3: Figure S2. Ramachandran plots of backbone dihedral angles for the three-dimensional structural models of PfVIT. [file 12936_2021_3827_MOESM3_ESM.pdf]

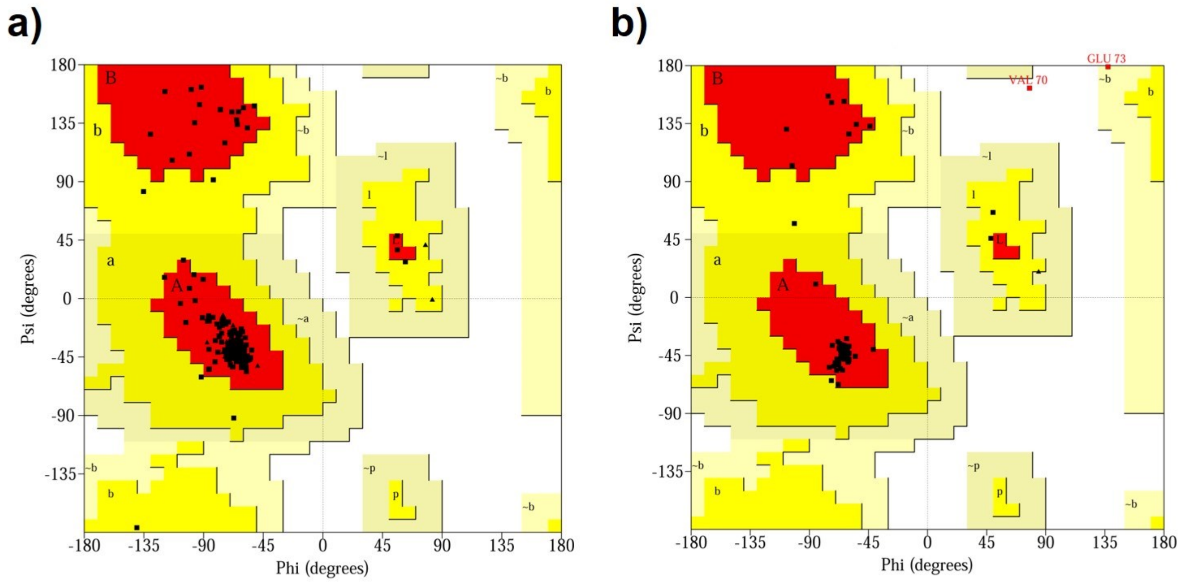

Figure S2: Ramachandran plots of backbone dihedral angles for the three-dimensional structural models of **(a)** the PfVIT protomer and **(b)** the isolated PfVIT cytoplasmic MBD. Individual amino acid residues are represented as black squares or triangles or (for residues in disallowed regions) as red squares. A, B and L represent the most favoured regions of the plot; a, b, l and p represent additionally allowed regions; ~a, ~b, ~l and ~p represent generously allowed regions. Disallowed regions are shaded in light yellow.
