# Supplementary material for: Characterization of the substrate binding site of an iron detoxifying membrane transporter from Plasmodium falciparum
Source: Malar J. 2021 Jun 30;20:295. doi: 10.1186/s12936-021-03827-7 (PMC8247066; doi:10.1186/s12936-021-03827-7)
Supplement: Supplementary file 4 — Additional file 4: Figure S3. Structural model of the functional PfVIT homodimer in an ‘inward open’ conformation and with Fe2+ substrate bound. [file 12936_2021_3827_MOESM4_ESM.pdf]

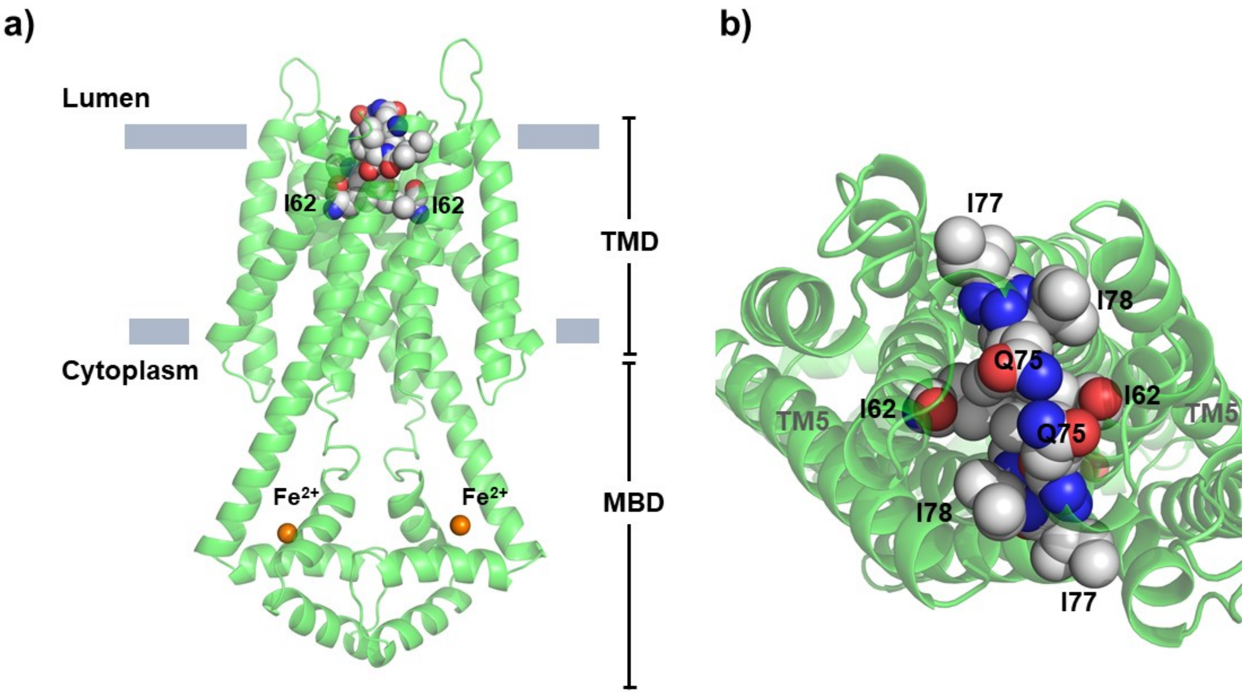

Figure S3: Structural model of the functional PfVIT homodimer in an ‘inward open’ conformation and with Fe^{2+} substrate bound. **(a)** PfVIT homodimer as viewed in the plane of the membrane. Amino acid residues that form the ‘plug’ in the substrate translocation pore at the lumenal side of the transmembrane domain (TMD) are represented in spacefill with oxygen atoms coloured red and nitrogen atoms coloured blue. Fe^{2+} ions bound to the cytoplasmic metal binding domain (MBD) of each PfVIT protomer are depicted as orange spheres. **(b)** The structural model viewed from the lumenal side of the membrane with amino acid residues that form the ‘plug’ represented in spacefill with oxygen atoms coloured red and nitrogen atoms coloured blue. For clarity, this view of the model was magnified x2.
